# Degradation of the Disease-Associated Prion Protein by a Serine Protease from Lichens

**DOI:** 10.1371/journal.pone.0019836

**Published:** 2011-05-11

**Authors:** Christopher J. Johnson, James P. Bennett, Steven M. Biro, Juan Camilo Duque-Velasquez, Cynthia M. Rodriguez, Richard A. Bessen, Tonie E. Rocke

**Affiliations:** 1 Prion Research Laboratory, United States Geological Survey National Wildlife Health Center, Madison, Wisconsin, United States of America; 2 Department of Veterinary Molecular Biology, Montana State University, Bozeman, Montana, United States of America; 3 Institute for Environmental Studies, University of Wisconsin, Madison, Wisconsin, United States of America; 4 Department of Bacteriology, University of Wisconsin, Madison, Wisconsin, United States of America; 5 Grupo de Investigación CENTAURO, Facultad de Ciencias Agrarias, Universidad de Antioquia, Medellín, Antioquia, Colombia; Creighton University, United States of America

## Abstract

The disease-associated prion protein (PrP^TSE^), the probable etiological agent of the transmissible spongiform encephalopathies (TSEs), is resistant to degradation and can persist in the environment. Lichens, mutualistic symbioses containing fungi, algae, bacteria and occasionally cyanobacteria, are ubiquitous in the environment and have evolved unique biological activities allowing their survival in challenging ecological niches. We investigated PrP^TSE^ inactivation by lichens and found acetone extracts of three lichen species (*Parmelia sulcata*, *Cladonia rangiferina* and *Lobaria pulmonaria*) have the ability to degrade prion protein (PrP) from TSE-infected hamsters, mice and deer. Immunoblots measuring PrP levels and protein misfolding cyclic amplification indicated at least two logs of reductions in PrP^TSE^. Degradative activity was not found in closely related lichen species or in algae or a cyanobacterium that inhabit lichens. Degradation was blocked by Pefabloc SC, a serine protease inhibitor, but not inhibitors of other proteases or enzymes. Additionally, we found that PrP levels in PrP^TSE^-enriched preps or infected brain homogenates are also reduced following exposure to freshly-collected *P. sulcata* or an aqueous extract of the lichen. Our findings indicate that these lichen extracts efficiently degrade PrP^TSE^ and suggest that some lichens could have potential to inactivate TSE infectivity on the landscape or be a source for agents to degrade prions. Further work to clone and characterize the protease, assess its effect on TSE infectivity and determine which organism or organisms present in lichens produce or influence the protease activity is warranted.

## Introduction

Transmissible spongiform encephalopathies (TSEs) are a group of infectious, neurodegenerative diseases including bovine spongiform encephalopathy, sheep scrapie, cervid chronic wasting disease (CWD) and human Creutzfeldt-Jakob disease (CJD) [1]. The etiological agent of TSEs appears to be primarily, if not exclusively, a misfolded isoform of the prion protein (PrP), termed PrP^TSE^ [2]. The PrP^TSE^ protein and TSE infectivity exhibit remarkable stability compared to typical pathogens and resist conventional decontamination methods such as autoclaving and disinfectants [3]. The resilience of TSE infectivity to inactivation has led to unexpected instances of disease transmission, such as CJD cases caused by contaminated surgical instruments subjected to conventional methods of cleaning and steam sterilization [4]. Scrapie and CWD differ from other TSEs in that epizootics can be maintained by horizontal transmission, as well as being mediated by an environmental reservoir of infectivity [5], [6]. Naïve sheep and deer have been infected following habitation in environments contaminated many years prior and, with a lack of evidence for vector-mediated transmission, environmental fomites have been implicated in the spread of these diseases [7]–[9]. Scrapie and CWD agents can likely enter the environment when shed from infected animals in saliva, urine or feces or when infected animals die on the landscape [10], [11]. The realization that TSE agents can remain infectious in the environment has led to the investigation of factors that could promote agent degradation and limit disease transmission. Microorganisms, proteases and manganese minerals have all been suggested to have the potential to reduce prion infectivity on the landscape or in engineered systems [12]–[14].

Lichens are symbiotic, plant-like associations between a fungi (mycobiont) and one, or occasionally more, photosynthetic partners (photobiont), such as a green alga or cyanobacterium species [15] and can contain internal bacterial communities [16], [17]. In present day ecosystems, including those inhabited by CWD-infected animals, lichens are ubiquitous, likely due to their early colonization of terrestrial environments [18]. Most lichens live on soil, bark, leaves or wood and can completely cover these surfaces [15]. Hostile environments, such as arctic tundra, deserts, bare rock surfaces or toxic slag heaps, are also habitable by lichens [19]–[24]. The ability of lichens with algal photobionts to fix carbon, or for species containing cyanobacteria to fix carbon and nitrogen, increases the range of niches that lichens can fill [25].

Lichens produce unique and unusual organic compounds that aid their survival and can have antibiotic, antiviral and other chemotherapeutic activities [26]. Over 800 of these compounds have been described and arranged into eight major groups: depsides, depsidones, dibenzofurans, usnic acids, anthraquinones, chromones, aliphatic acids, and pulvinic acid derivatives [25], as well as many minor groups [26]. The production of other types of biomolecules by lichens remains less well characterized, but an expanding literature indicates that lichens produce metabolic enzymes, antioxidant enzymes [27], laccase activity [28], [29], catalase-like enzymes [30] and possibly proteolytic enzymes [31], [32].

In this study, we investigated the ability of lichens to degrade PrP^TSE^ *in vitro* in order to assess the potential that lichens inactivate prions in the environment. The rationale for this approach is that the unique biology and diverse biosynthetic capabilities of lichens may allow these organisms to produce molecules capable of inactivating or degrading prions. In the current study, we report that lichen extracts and intact lichens can degrade PrP^TSE^ *in vitro* and the effect is mediated by a serine protease.

## Results

### Degradation of PrP by lichen extracts

**Figure 1:**
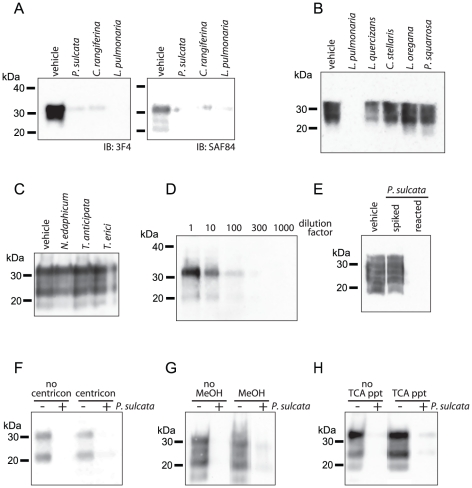
Extracts of the lichens *P. sulcata*, *C. rangiferina* and *L. pulmonaria* degrade PrP. Equivalent amounts of PTA-enriched PrP^TSE^ (30 µg total protein) were incubated with the indicated lichen extracts (labels, 10 mg lichen equivalents) or with vehicle and were subjected to NuPAGE and immunoblotting. Extracts of lichens in panel (A) reduced PrP immunoreactivity compared to control, whereas extracts of lichens in panel (B) do not reduce PrP immunoreactivity. (C) Treatment of PrP^TSE^ using the method employed in panels (A) and (B) with extracts of isolated algae or cyanobacterium cultures (10 mg equivalents) do not degrade PrP. (D) Dilutions of control reactions containing PrP^TSE^ and no lichen extract indicate that PrP immunoreactivity was no longer detectable when dilution factors were greater than 100. (E) Adding *P. sulcata* extract to PrP^TSE^, but not allowing it time to react (spiked) did not reduce PrP immunoreactivity compared to control (vehicle). Equivalent samples in which *P. sulcata* extract was allowed time to incubate (reacted) did not have detectable PrP immunoreactivity. (F–H) Methods to remove lichen compounds and DMSO from samples post-reaction, such as (F) washing remaining protein with PBS using a centricon filter column (10–12 kDa MWCO) or (G & H) precipitating protein using methanol (MeOH) or trichloroacetic acid (TCA) do not restore PrP immunoreactivity. All immunoblots (IB) used anti-PrP mAb 3F4, except for panel (A) which used both 3F4 and SAF84.

Preparations enriched for PrP^TSE^ using phosphotungstic acid (PTA) were treated with lichen extracts or with vehicle as a control, then examined by immunoblotting using PrP-specific antibodies 3F4 and SAF84. Extracts of the lichens *Parmelia sulcata*, *Cladonia rangiferina* and *Lobaria pulmonaria* all reduced the amount of PrP detected in immunoblots using either antibody (Figure 1A). The reduction in PrP immunoreactivity by lichen extracts appears to be species-specific, as extracts of lichens from the same genera (*P. squarrosa*, *L. quercizans*, *L. oregano* and *C. stellaris*), were not able to substantially reduce PrP levels (Figure 1B). Lichens are classified by the taxonomy of the mycobiont and within the same genera, lichens commonly have similar or identical photobionts [15]. We tested extracts of isolated lichen photobiont algae (*Trebouxia anticipata* or *T. erici*) or cyanobacterium (*Nostoc edaphicum*), but found they were unable to degrade PrP (Figure 1C), suggesting that the fungal partners of lichens may be responsible for activity.

**Figure 2:**
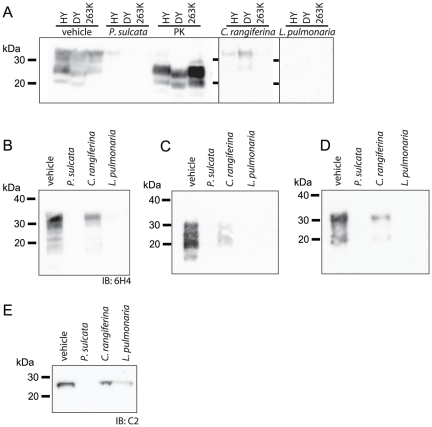
Lichen extracts degrade PrP^TSE^ in brain homogenate. (A) Lichen extracts (10 mg lichen equivalents) were incubated with equal amounts (10 µL) of 10% brain homogenates from hamsters infected with Hyper (HY) TME, Drowsy (DY) TME or 263K scrapie strains of TSE agents and PrP degradation was assessed by immunoblotting. Treatment of each hamster brain homogenate with 50 µg·mL^−1^ of PK demonstrates the presence of abnormal PrP in the starting material. Using the same conditions as in (A), lichen extracts cause degradation of PrP in (B) a CWD-infected white-tailed deer, (C) PK-treated HY hamster and (D) uninfected hamster brain homogenates. (E) Protein C5 of the 20S subunit of the proteasome, an unrelated protein, was also degraded by lichen extracts. Immunoblots (IB) used mAbs 3F4 (A, C and D), 6H4 (B) or pAb anti-C5 (E).

**Figure 3:**
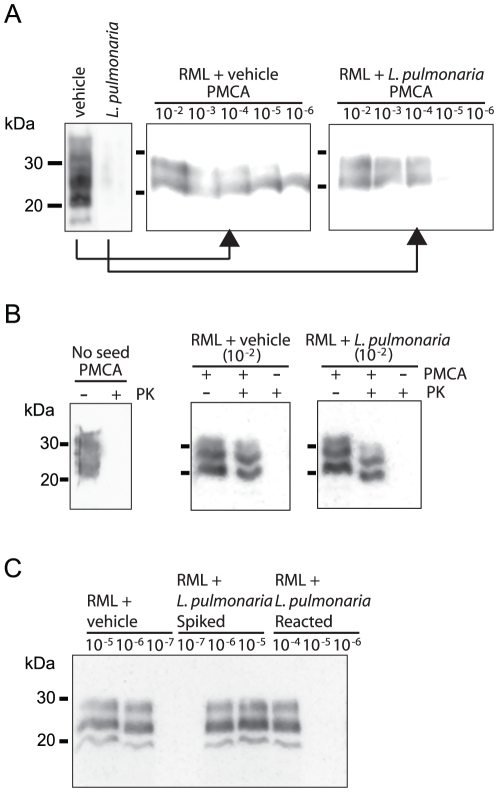
Semi-quantitative PMCA to assess RML PrP^TSE^ degradation. (A) The extent of mouse RML PrP^TSE^ degradation was measured by PMCA analysis of dilutions (10^−2^–10^−6^) of vehicle or *L. pulmonaria* extract-treated RML. (B) Control reactions lacking PrP^TSE^ seed or sonication did not show amplification as measured by PK digestion. (C) RML samples were incubated with vehicle for 1 hr or with *L. pulmonaria* extract (reacted: 1 hr; spiked <10 sec) then diluted and subjected to PMCA. Adding *L. pulmonaria* extract in PMCA reactions without allowing time for reaction (spiked samples) does not appear to reduce PrP^TSE^ levels or affect amplification compared to control (vehicle). All immunoblots used mAb SAF83.

**Figure 4:**
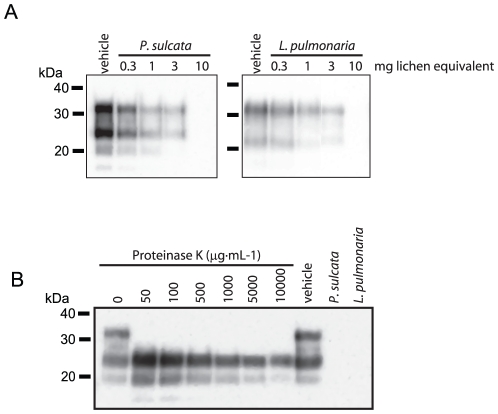
Effect of lichen extract dose on PrP degradation. (A) Dose-response of the indicated lichen extracts (0–10 mg lichen equivalents) on degrading PTA-enriched PrP^TSE^ (30 µg total protein). (B) The indicated concentrations of PK (0–10000 µg·mL^−1^) were incubated with PTA-enriched PrP^TSE^ (30 µg total protein) to assess degradation and for comparison with lichen extracts. Immunoblots used mAb 3F4.

**Figure 5:**
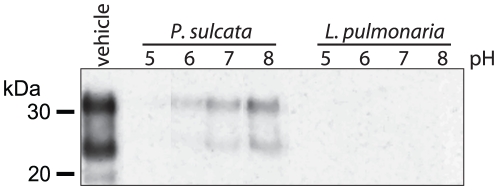
Effect of pH on PrP degradation by *P. sulcata* and *L. pulmonaria* extracts. Reaction solutions were buffered at the indicated pHs (5–8) and the effect of each indicated lichen extract (10 mg lichen equivalents) on degradation of PTA-enriched PrP^TSE^ (30 µg total protein) was observed by immunoblotting with mAb 3F4.

**Figure 6:**
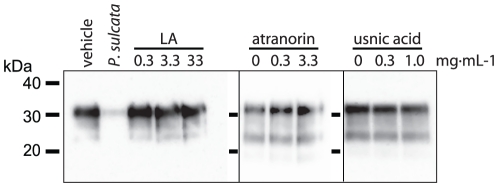
Common lichen chemicals do not affect PrP levels. Samples of PTA-enriched PrP^TSE^ (30 µg total protein) were incubated with the indicated concentrations of lecanoric acid (LA), usnic acid, atranorin or vehicle followed by immunoblot analysis with mAb 3F4. *P. sulcata* extract (10 mg lichen equivalent) served as a positive control for PrP degradation.

**Figure 7:**
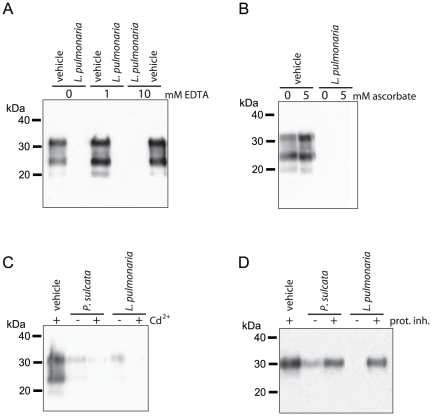
Assessing enzymatic activity responsible for PrP degradation. Reactions of PTA-enriched PrP^TSE^ (30 µg total protein) and the indicated lichen extracts (10 mg lichen equivalents) were prepared with enzyme inhibitors: (A) EDTA (1 or 10 mM) to inhibit metalloenzymes, (B) ascorbate (5 mM) as an antioxidant and chelator, (C) Cd^2+^ (80 mM) to inhibit laccases or (D) a broad-spectrum protease inhibitor cocktail (10 µL per reaction). The effect of each inhibitor on *P. sulcata* or *L. pulmonaria* extract-mediated degradation of PrP was measured by immunoblotting with mAb 3F4.

**Figure 8:**
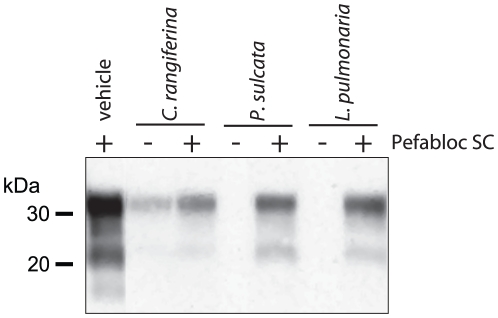
The serine protease inhibitor Pefabloc SC prevents PrP degradation. Incubation of PTA-enriched PrP^TSE^ (30 µg total protein) with lichen extracts (10 mg lichen equivalents) were performed in the presence and absence of Pefabloc SC (14 mM). Reactions with the inhibitor had increased PrP immunoreactivity with mAb 3F4 (reduced PrP degradation).

**Figure 9:**
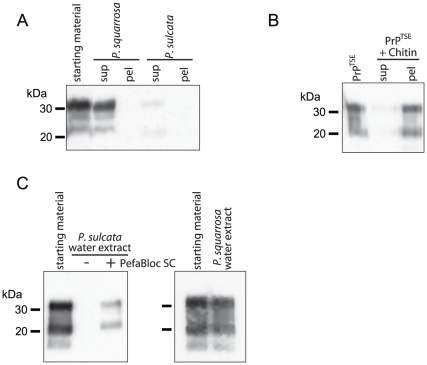
Intact *P. sulcata* tissue or a water extract of the lichen degrades PrP. (A) Freshly-collected *P. sulcata* or *P. squarrosa* (4 mg, each) were incubated with infected brain homogenate (100 µL of 10% v/w in distilled water) for 24 h. Following incubation, the lichen-treated brain homogenates (sup) and the lichen tissues (pel) were analyzed for PrP by immunoblotting with mAb 3F4. A reduction in PrP signal was associated with *P. sulcata*, but not with *P. squarrosa*. (B) PTA-enriched PrP^TSE^ (30 µg total protein) exposed to chitin beads is bound by the beads, but is extractable using NuPAGE sample buffer. Little immunoreactivity in the supernatant (sup) shows the protein binds chitin and treatment of bound PrP with sample buffer yields immunoreactivity approximately equal to PrP starting material. (C) A water extract from *P. sulcata*, but not *P. squarrosa*, causes PrP degradation. Immunoblotting comparison of PTA-enriched PrP^TSE^ (30 µg total protein) incubated in only water or exposed to a water extract of *P. sulcata* (100 mg lichen equivalent) with and without the serine protease inhibitor Pefabloc SC (10 mM). Water extract of *P. sulcata* induces a substantial reduction of immunoreactivity that is blocked by PefaBloc SC whereas a water extract of the related lichen *P. squarrosa* does not degrade PrP under the same conditions.

The remaining amount of immunoreactivity following treatment of PrP^TSE^ with *P. sulcata*, *C. rangiferina* or *L. pulmonaria* extracts, throughout the array of experiments performed here, ranged from no detectable PrP to a slight band remaining (Figures 1, 2, 3, 4, 5, 6, 7, 8, and 9). In Figure 1A, we present a conservative representation of the amount of PrP signal lost: slight bands are seen in most lanes. Often, however, we observe no remaining immunoreactivity when treating PrP^TSE^ with these lichen extracts. Because lichen extract-treated PrP^TSE^ samples are frequently below the detection limit of our assay, we immunoblotted a dilution series of PrP^TSE^ (Figure 1D) to better quantify the detection limit of our procedures [33], [34]. Dilutions of control samples identical to those in Figure 1A and B (containing PrP^TSE^ and vehicle, no lichen extract) were detectable by immunoblotting until the dilution was increased beyond a factor of 100. These data indicate that in samples where no PrP was detected following lichen treatment, PrP levels were reduced by at least two log units.

To control for the possibility that lichen materials in the extracts inhibited protein separation, transfer or immunodetection, we compared samples of PrP^TSE^ that had been reacted with *P. sulcata* extract for 1 hr to samples that the extract was spiked into immediately prior to processing (Figure 1E). As was seen in Figure 1A, samples in which *P. sulcata* extract was allowed to react with the PrP^TSE^ had substantially reduced PrP immunoreactivity, whereas there was no loss of signal when the extract was spiked in and samples were immediately processed (Figure 1E). We tried additional measures to restore PrP levels (Figure 1F–H). We attempted to recover immunoreactivity in lichen extract-treated samples by filtering reactions through a 10–12 kDa molecular weight cutoff (MWCO) membrane and washing reactions with 50 volumes of phosphate buffered saline (PBS). Lichen extract-treated samples did not regain immunoreactivity following washing and vehicle-treated samples had similar amounts of immunoreactivity whether washed or not (Figure 1F). Similarly, precipitating protein following reactions with lichen extracts using ice-cold methanol or tricholoracetic acid did not restore the immunoreactivity lost from lichen-treated samples (Figure 1G and 1H). These data support the interpretation that the lichen extracts are degrading PrP^TSE^ and not interfering with detection.

### Lichen extracts degrade PrP^TSE^ in brain homogenate (BH)

Enriched PrP^TSE^ may not have identical physicochemical properties to PrP^TSE^ in the brain and different strains of TSE agents, such as Hyper (HY) transmissible mink encephalopathy (TME), Drowsy (DY) TME and 263K scrapie, have varying susceptibly to proteolytic degradation by the serine protease, proteinase K (PK) [35], [36]. We tested the ability of three lichen extracts to degrade these three strains of hamster TSE agent in BH (Figure 2A). Each lichen extract diminished PrP levels in BH from animals infected with each strain of agent and some were reduced below the limit of immunoblotting detection. Low levels of immunoreactivity remain post-incubation in the HY samples treated with *P. sulcata* extract, whereas DY and 263K are degraded below the threshold of detection. Both HY and DY samples treated with *C. rangiferina* extract retain some immunoreactivity while 263K was not detected. Extract of *L. pulmonaria* reduced PrP below the limit of detection for each strain. Degradation of PrP by lichen extracts in infected BH samples or PrP^TSE^-enriched preps differs from digestion by PK in that PK cleaves an *N*-terminal portion of the PrP^TSE^ molecule, leaving a truncated end-product, whereas lichen extracts can reduce PrP immunoreactivity to below the limit of immunoblotting detection. Similar to these findings, each extract reduced levels of PrP in CWD-positive deer BH (Figure 2B); CWD BH samples treated with *P. sulcata* and *L. pulmonaria* no longer possessed PrP immunoreactivity whereas some, but not all immunoreactivity was lost from *C. rangiferina* treated samples.

The PrP in an infected brain exists as a mixture of both PrP^TSE^ and cellular prion protein (PrP^C^) [37]. We assessed whether lichen extracts are capable of degrading both isoforms of PrP by incubating PK-treated, HY-infected BH, which contains only the disease-associated PrP isoform, PrP^TSE^ (Figure 2C) or healthy hamster BH, which contains only PrP^C^ and no PrP^TSE^ (Figure 2D) with *P. sulcata*, *C. rangiferina* and *L. pulmonaria* extracts. We found that both disease-associated and normal isoforms of PrP could be degraded by these extracts. Similar to Figure 2A, we found complete degradation of both isoforms by *P. sulcata* and *L. pulmonaria* extracts and some remaining immunoreactivity in samples treated with *C. rangiferina* extract.

The degree to which a lichen extract degraded PrP did not necessarily correspond to its activity toward degrading another unrelated protein in hamster BH (Figure 2E). Following lichen extract incubation, protein C2 of the 20S subunit of the proteasome was no longer detectable in *P. sulcata* extract-treated samples, but was detected in *C. rangiferina* and *L. pulmonaria*-treated BH. In contrast, *L. pulmonaria* extract was highly effective at degrading PrP in infected BH (Figure 2A and C) and PrP^C^ in uninfected BH (Figure 2D).

Trace amounts of PrP^TSE^ can be quantified using protein misfolding cyclic amplification (PMCA), a procedure that uses sonication to cause PrP^TSE^ to template conversion of PrP^C^ to the infectious form of the protein. To assess the extent of PrP^TSE^ degradation in BH of RML strain-infected mice by lichen extracts, we subjected dilution series of vehicle and *L. pulmonaria*-treated BH to a single round of amplification by PMCA (Figure 3A). We were able to amplify PrP^TSE^ from vehicle-treated BH at dilutions 10^−2^ to 10^−6^, but not at 10^−7^. In BH treated with *L. pulmonaria* extract, we could not detect amplification in samples with dilution factors greater than 10^−4^, indicating at least a 100-fold loss of prion templating activity. Controls in which no PrP^TSE^ was added or which were not subjected to sonication did not show amplification (Figure 3B). We also assessed whether lichen extract, even though at trace levels in PMCA samples, would inhibit PMCA (Figure 3C). Samples of RML BH were treated with either vehicle or *L. pulmonaria* extract for 1 hr or were spiked with *L. pulmonaria* extract and immediately processed for PMCA by dilution into PMCA buffer. No differences in amplification were observed in vehicle-treated or lichen extract spiked samples, whereas amplification was not observed in *L. pulmonaria*-treated BH at dilutions greater than 10^−4^. These data are consistent with our immunoblotting results that show lichen extracts cause at least a two log reduction in PrP levels.

### Dose and pH-dependence of PrP degradation by lichen extracts

We examined the dose-dependence of *P. sulcata* and *L. pulmonaria* extract-mediated PrP degradation. These lichen extracts appear to reduce PrP immunoreactivity to a larger extent than does the *C. rangiferina* extract. Samples PTA-enriched for PrP^TSE^ were incubated with extracts equivalent to 300 µg to 10 mg of lichen starting material (Figure 4A). For both lichen extracts, dose-dependent losses of PrP immunoreactivity were observed. The highest dose tested of each lichen extract (10 mg lichen equivalent) was sufficient to reduce immunoreactivity below the threshold of detection.

We attempted to identify a concentration of PK that would reduce PrP immunoreactivity to the same degree as the lichen extracts by incubating PrP^TSE^ with doses of PK from 50 to 10,000 µg·mL^−1^. We found that under identical time and temperature conditions (1 h at 37°C), and in solution buffered to pH 7.0, which favors PK activity, no tested dose of PK reduced PrP immunoreactivity below the limit of detection (Figure 4B). Comparing PrP^TSE^ samples treated with 50 and 10,000 µg·mL^−1^ of PK using densitometry, we found that treatment with 10,000 µg·mL^−1^ reduced immunoreactivity by 73.3% in the blot shown, whereas both immunoblotting and PMCA experiments (Figures 1D and 3A, respectively) indicated a greater loss of PrP immunoreactivity or PMCA activity when treated with lichen extract.

We also examined the effect of pH on degradation of enriched PrP^TSE^ by these extracts (Figure 5). Unbuffered reactions were found to be at pH 5.6. Over the buffered range from pH 5.0–8.0, degradation of PrP by *P. sulcata* extract was enhanced in reactions buffered to acidic conditions compared to reactions buffered at pH 8.0. Notably, PrP degradation by *L. pulmonaria* extract was equally effective from pH 5.0 to 8.0 (Figure 5) and did not exhibit pH-dependence at a wider range of pH 4–11 values or when one-tenth the amount of *L. pulmonaria* extract was used over the pH range 4–8 (data not presented). When taken together, these data suggest concentration and pH can influence *P. sulcata*-mediated PrP^TSE^ degradation whereas *L. pulmonaria* extract functions at a wider pH range.

### Discerning the Mechanism of PrP Degradation: Lichen Chemistry and Compounds

Lichens produce a myriad of organic chemical compounds, some with unusual activities [26]. The chemicals found in individual lichen species are fairly well known, as they are used for taxonomical purposes. We compared the known compounds in *P. sulcata*, *C. rangiferina* and *L. pulmonaria*, as well as those species found to not degrade PrP (Figure 1B), but did not find a correlation between PrP degradation and lichen chemistry. Despite not identifying obvious lichen compound targets, we incubated PrP^TSE^ with three common lichen secondary metabolites at varying concentrations to assess whether they would reduce PrP immunoreactivity (Figure 6). We used atranorin, lecanoric acid, and usnic acid because they are among the most common lichen secondary metabolites and are found in the species we studied. The first two compounds are depsides, while usnic acid is a dibenzofuran, and all are uniquely produced in lichens. We incubated PrP^TSE^ with concentrations of these chemicals equal to, or higher than, those found in lichen extracts. The highest doses of each that we tested were near the limit of solubility of each chemical. We observed that none of the tested lichen secondary metabolites affected PrP levels in our immunoblots indicating that they neither degraded PrP^TSE^ nor caused interference with the assay.

### Discerning the Mechanism of PrP Degradation: Enzymes

Given no clear evidence that lichen secondary metabolites alone are responsible for the loss of PrP from lichen extract-treated samples, we hypothesized that degradation was due, at least in part, to enzymatic activity. We tested whether degradation of PTA-enriched PrP^TSE^ could be blocked with inhibitors of various classes of degradative enzymes found in lichens or fungi [29], [30], [38] (Figure 7). The increase in amount of PrP immunoreactivity over signal found in extract-treated samples lacking inhibitor would indicate the degree to which the inhibitor prevented PrP degradation.

The chelator EDTA interferes with the activity of metalloenzymes found in fungi such as peroxidases and laccases. Neither 1 nor 10 mM EDTA prevent degradation of PrP by *L. pulmonaria* extract (Figure 7A). Similarly, to limit redox reactions and inhibit some metalloenzymes, we used ascorbate, an antioxidant and chelator. We found ascorbate (5 mM) did not affect the degradation of PrP by *L. pulmonaria* extract (Figure 7B) To inhibit laccases, we added Cd^2+^ (80 mM, final concentration) [39] to reactions of PrP^TSE^ with either *P. sulcata* or *L. pulmonaria* extract, but did not observe inhibition of PrP degradation (Figure 7C). Finally, to test whether degradation was due to protease activity, we used a protease inhibitor cocktail designed to block many classes of proteases (Figure 7D). We found that this inhibitor preserved some PrP immunoreactivity in samples treated with either *P. sulcata* or *L. pulmonaria* extracts, suggesting that one or more proteases are responsible for degradation. Additionally, we tested a second protease inhibitor cocktail from a different manufacturer (Roche) and observed similar results (data not presented).

To identify the class of protease responsible for degrading PrP, we screened each of the ten inhibitors included in the Roche protease inhibitor cocktail for the capacity to prevent PTA-enriched PrP^TSE^ degradation. The compounds and concentrations used are presented in Table 1. We found that only the serine protease inhibitor, Pefabloc SC, limited PrP^TSE^ degradation in samples treated with *P. sulcata*, *C. rangiferina* or *L. pulmonaria* extracts (Figure 8). Not all starting immunoreactivity is retained in lichen extract-treated samples containing Pefabloc SC, and the effect is more prominent on *P. sulcata* and *L. pulmonaria* extracts than on *C. rangiferina* extract. The inability of other protease inhibitors to block PrP degradation and the efficacy of Pefabloc SC suggests that a serine protease is involved in the degradation of PrP by lichen extract. Our results, however, do not exclude the possibility that other components of the lichen extract could act synergistically to degrade PrP.

**Table 1:** Protease inhibitors tested to interfere with PrP degradation by lichen extracts.

Individual Protease Inhibitors	Tested Doses
Pepstatin A	0.1–100 µM
Antipain-dihydrochloride	10 mM
Bestatin	1 mM
Chymostatin	1.8 mM
E-64	18 mM
Leupeptin	500 µM
Phosphoramidon	1 mM
Aprotinin	50 µM
Pefabloc SC*	1–50 mM

*Only Pefabloc SC inhibited degradation of PrP by lichen extracts.

### Degradation of PrP by Intact Lichen Tissue

The previous experiments utilized lichen extracts, and while they indicate that lichens possess protease activity capable of degrading PrP^TSE^, they do not represent or simulate environmental conditions in which an intact lichen organism could be in direct contact with prions in the environment. To examine whether lichens could degrade PrP under more relevant conditions, we incubated intact, freshly-collected *P. sulcata* and *P. squarrosa* in solutions of dialyzed HY-infected hamster BH for 24 h at 20°C and measured PrP levels in BH and lichen tissue (Figure 9A). Our data show that extracts of *P. sulcata* cause degradation of PrP whereas extracts of *P. squarrosa* do not (Figure 1A and B). We found that following incubation, PrP levels were reduced in BH supernatants exposed to *P. sulcata*, compared to BH incubated with *P. squarrosa* or in the absence of lichen. We tested whether either lichen tissue accumulated PrP and thereby removed it from the supernatant. No PrP immunoreactivity was detected in either lichen tissue pellets following homogenization and heating in NuPAGE lithium dodecyl sulfate (LDS) sample buffer with reducing agent. As a control for recovery of PrP from lichen tissues, of which chitin is a major component, we tested whether PTA-enriched PrP^TSE^ sorbed to chitin-coated beads could be removed by heating in LDS sample buffer (Figure 9B). We found that the PrP was bound by chitin, but that LDS sample buffer was sufficient to remove most or all of the bound protein. These data suggest that *P. sulcata* tissue is capable of degrading PrP^TSE^.

Using freshly-collected *P. sulcata* obtained from two separate locations approximately 280 km apart, we incubated fresh lichens with infected BH to assess whether collection site would influence PrP degradation by *P. sulcata*. We found that lichens collected at both sites were capable of degrading PrP and we compared the remaining PrP immunoreactivity from lichen-treated samples with non-treated samples (data not presented). There was no significant difference in effect between *P. sulcata* collected at either location (average loss of immunoreactivity of 90±10% for Wisconsin Dells and 80±12% for Eagle River; t = −0.8, df = 14, p = 0.45). The similarity of these values suggested that geographic location may not affect the capability of *P. sulcata* to degrade PrP^TSE^, at least in this study. It is worthwhile to note that CWD-infected deer have been harvested from the same Wisconsin Dells area as the lichen used in this study was collected. The data in Figure 9A are from lichens collected at the Eagle River site.

As PrP was degraded by intact *P. sulcata* tissue, we hypothesized that the protease could be leached from the lichen and act on the protein in solution. We, therefore, generated water extracts of *P. sulcata* and *P. squarrosa* and tested whether they would cause degradation of PTA-enriched PrP^TSE^ (Figure 9C). We found that, compared to water alone, PrP levels were reduced when incubated in the *P. sulcata* aqueous extract, but that the *P. squarrosa* extract fails to cause PrP degradation (Figure 9C), as we had seen in the acetone extracts (Figure 1A and B). We also found that the serine protease inhibitor PefaBloc SC prevented complete degradation of PrP by *P. sulcata*, consistent with water extracts containing the same anti-PrP activity found in the lichen organic extract (Figure 8).

## Discussion

The identification of factors in the environment that degrade prions is critical to our ability to control and mitigate the effects of scrapie and CWD and understand the fate of prions in natural environments. In this study, we found that extracts of three lichen species have a serine protease activity capable of reducing PrP levels in PrP^TSE^-enriched preps or infected BH *in vitro*. In experiments more similar to environmental conditions, we tested one of those lichen species and found that intact lichen tissue or a water extract of the tissue could also degrade PrP. The speculated protein-only nature of the TSE agent has prompted numerous studies of proteases as potential prion decontaminants [40]–[45] and has prompted investigation into their use in soil environments [46]. Typical conditions used for prion inactivation by proteases, however, involve elevated temperatures, the presence of detergents and extreme pH values.

Our discovery that lichens contain proteases capable of degrading PrP under mild conditions or when lichens are simply exposed to infected BH *in vitro* contributes to the repertoire of potential prion decontaminants. While great caution must be exercised in extrapolating *in vitro* studies to environmental conditions, our data suggest lichens could contribute to prion degradation on the landscape. If this were the case, inactivation of prions could occur by direct contact of agent with lichens or perhaps in soil near lichens. The amount of lichen protease present in the environment is challenging to estimate and is likely to depend on its own environmental stability and its propensity to be leached from lichens. In our study, the quantity of lichen acetone extract used in reactions represents ≤10 mg of intact lichen tissue, a small fraction of a typical specimen, and our findings may underrepresent the ability of lichens to degrade prions shed directly onto lichen surfaces. The extractability of proteases from lichens in the environment is unknown, but our data show that a water extract of *P. sulcata* contains prion-degrading protease activity (Figure 9C) and suggest the potential of its presence in lichen leachate. Previous work has indicated that water is a suboptimal extractant for lichens [28], [47], [48] and our own studies indicate that ten-fold more lichen tissue is needed to produce an aqueous extract with protease activity than with acetone. Despite limited extractability by water, lichen chemicals can be found in leachates and soil; usnic acid, for example, was found in soil under lichens at 1–4 ppm [49]. Should the lichen protease be present at similar or higher levels, and be active, it is conceivable that lichen leachates could influence the survival of prions in the environment.

When treated with lichen extracts, even at doses not sufficient to degrade all PrP, PrP^TSE^ does not appear to undergo the *N*-terminal cleavage observed with PK treatment (Figures 2 and 3). Analysis of residual PrP immunoreactivity following lichen extract treatment using antibodies to various epitopes capable of recognizing PrP^TSE^ from multiple species suggests degradation of PrP, rather than alteration of the protein to a form unrecognized by a particular antibody. Additionally, inhibition of PrP degradation by PefaBloc SC, a serine protease inhibitor, suggests that proteolysis is a major factor responsible for PrP degradation by lichen extracts, but may not be the only factor. It is noteworthy that PK, another serine protease, even at high concentrations, like 1.6 mg·mL^−1^ [50] or 10 mg·mL^−1^ (Figure 4B), was not capable of degrading PrP^TSE^ to the same extent as the crude lichen extract we examined (Figure 3B). Previous work suggests that longer incubation periods may be necessary for PK to degrade PrP^TSE^ to the same degree that we have found with lichen extracts [51], [52]. Purified or concentrated lichen protease could be expected to promote even more PrP^TSE^ degradation than lichen extracts, but further investigation is needed to identify if other components or enzymes in the lichen extracts may synergize with the serine protease to promote PrP^TSE^ degradation.

Our current investigation has focused on the degradation of PrP^TSE^ and, clearly, investigation into the effect of lichen proteases on TSE infectivity is a critical next step. Studies have observed dramatic losses in PrP levels but with minimal effect on infectivity [53], [54]. Conversely, lichen extracts may diminish prion infectivity by affecting prion stability, structure or replication without reducing the total amount of PrP [55]. The PrP degradation activity observed in each lichen extract may reflect the quantity of protease present or amounts of required cofactors; the contribution of lichen secondary metabolites to the degradation of PrP remains unclear. Lichen organic, and potentially water, extracts can be rich in secondary metabolites that often have potent activities [56]–[58]. While, our findings suggest that a serine protease is a major contributor to PrP degradation, our data does not exclude the possibility that lichen secondary metabolites may function as cofactors for lichen enzymes or even sensitize PrP to protease digestion.

The pH-dependence of *P. sulcata* extract and the unaltered PrP degradation by *L. pulmonaria* extract across a wide range of pH values points to the existence of differences in proteolytic mechanisms among lichen species. The specificity of whether or not a given lichen species extract can degrade PrP appears to be due to the mycobiont component of the lichen, as extracts of close relatives of *P. sulcata*, *C. rangiferina* and *L. pulmonaria* with the same or similar photobionts did not display this activity. Identical lichen species collected at different times or locations performed identically with respect to PrP degradation, further supporting the concept of species specificity.

We were able to identify only two other studies concerned with proteases in lichens. Shapiro *et al.* detected protease activity in six lichen species by amine production and compared it with nitrogenase activity in order to evaluate protein synthesis and degradation [32]. No information was provided in this study regarding whether photobionts or mycobionts were responsible for the protease activity. Avalos *et al.* found evidence indicating photobiont hydrolase activation in the lichen *Evernia prunastri* [31]. Our examination of extracts from common lichen photobionts suggests that the proteolytic activity in our extracts could be due solely to the mycobionts. The lack of PrP degrading activity in related species that possess similar or identical photobionts (Figure 1B) further supports the role for the mycobiont in PrP degradation. Our data does not exclude, however, that photobionts living in symbiosis with the mycobiont may behave differently than the free-living organisms. In both the photobiont and the mycobiont, symbiosis would be expected to differentially regulate the production of various gene products and may affect the production of the proteolytic activity capable of degrading PrP. An expanding literature suggests that lichens can host a range of bacterial species that impact growth and metabolic characteristics of the myco and photobionts [16], [17]. Our data also do not exclude the possibility that lichen-associated bacteria could contribute to the degradation of PrP and further investigation into what organism is contributing the degradative activity is needed.

The question of why a lichen protease would be capable of degrading PrP^TSE^ is intriguing. Yeast can be infected by a number of fungal prions with different amino acid sequences than mammalian prions [59], but lichen mycobionts have not, to our knowledge, been examined as potential hosts. Mammalian and fungal prions share a common amyloid structure [60], suggesting that lichen proteases may have activity against fungal prions as well. The presence of an enzyme capable of degrading PrP^TSE^ in lichens could suggest protection against prions or amyloids favors some aspect of lichen survival.

Interactions between lichens and wildlife are well documented [61]. The most well known examples are reindeer foraging on lichen mats in the boreal zone and the recent elk mortality events due to consuming toxic amounts of lichens in Wyoming [62]. It is not unreasonable to hypothesize that dietary lichens could affect CWD transmission or pathogenesis in cervids. Our data argue that investigation of these putative interactions is warranted, as is further characterization of the mechanism of PrP degradation by lichens, including identification of responsible enzymes and any potential cofactors.

## Materials and Methods

### Lichens, Isolated Photobionts and Extracts

The lichen species selected for study were: *Parmelia sulcata* from Isle Royale, Michigan (collected in 1999) or from Wisconsin Dells or Eagle River, Wisconsin (collected in 2008 or 2009, respectively); *P. squarrosa* from the Superior National Forest (NF), Minnesota (1999) or from Eagle River Wisconsin (collected in 2009 and 2010); *Cladonia rangiferina* from Isle Royale (1999); *C. stellaris* from Superior NF (1999); *Lobaria pulmonaria* and *L. quercizans* from Superior NF (1999), and *L. oregana* from the Oregon coast (2002). These species differ in morphology, myco- and photobionts and habitat: *Parmelia* spp. are often found growing on trees and rocks, *Cladonia* spp. are a common ground cover and *Lobaria* spp. typically grow on trees. Lichens were preserved at room temperature either in a desiccated and stable dormant state in opaque, breathable bags from the date of collection or as a ground powder in plastic bags.

Dried lichens were powdered using a Retsch mixer mill MM 200 (Newtown, PA) in polytetrafluoroethylene grinding jars. Extracts were produced by suspending lichen powders in acetone at 10% (w/v) and incubating at 37°C for 24 h with vigorous shaking. Following incubation, solid particles were removed by filtration through Whatman filter paper (Grade #1, Piscataway, NJ), and the acetone in the filtrate was evaporated. The remaining residue was resuspended in dimethyl sulfoxide (DMSO) (ThermoFisher Scientific, Rockford, IL) such that each g of lichen powder starting material would yield 1 mL of product.

Isolated lichen photobionts (*Nostoc edaphicum*, *Trebouxia anticipata* and *T. erici*) were purchased from the UTEX Culture Collection of Algae (University of Texas, Austin, TX) and were grown as agar cultures according to directions supplied by the Culture Collection. Following 8 weeks of growth, photobionts were collected and powdered using liquid nitrogen. Extracts were produced by suspending powders in acetone at 10% (w/v) and processing as described earlier for production of lichen extracts.

Water extract of *P. sulcata* or *P. squarrosa* was prepared by placing 1 g of intact lichen into 10 mL of deionized water and shaking for 24 h at 20°C. Following incubation, particles were removed by centrifugation at 5000 *g* for 5 minutes and water extracts were collected by pipette.

### Source of Prion Protein and brains

All animal work was conducted with approval of the National Wildlife Health Center institutional animal care and use committee (Protocol #EP080716). Hamster PrP^TSE^ was generated by the experimental infection of Syrian golden hamsters with the HY or DY strains of hamster-passaged TME agent or 263K strain of hamster-passaged scrapie [63], [64]. The HY strain of TME from hamsters is used in all experiments unless another specific agent is listed. Uninfected brains were from animals never experimentally challenged or exposed to areas where prion-challenged animals were housed. White-tailed deer CWD agent was obtained from the obex of a hunter-harvested G96 *Prnp* genotype (GenBank accession number AF156185) animal [65], [66]. The RML strain of mouse-passaged scrapie was generated by experimental infection of CD-1 mice.

Brain homogenate (BH) was made by adding brain tissue to a final concentration of 10% (w/v) in PBS and homogenizing in a Dounce homogenizer. Some experiments used BH that was dialyzed using Slide-A-Lyzer cassettes (10–12 kDa MWCO; ThermoFisher Scientific) against three changes of deionized water over a 24-h period. Proteinase-K (PK)-treated BH was generated from 10% HY-infected BH treated with 50 µg·mL^−1^ PK (Promega, Madison, WI) for 1 hr at 37°C. Activity of PK was blocked with 1 mM PefaBloc SC (Roche Applied Science, Germany) on ice and then frozen prior to use. Phosphotungstic acid (PTA)-enriched PrP^TSE^ preparations were produced using published methods [67]. Briefly, 4% PTA (Sigma-Aldrich, St. Louis, MO) in a 170 mM MgCl_2_, pH 7.4 solution was added to 5% BH in 2% sarkosyl to a final concentration of 0.25% PTA. Homogenate was incubated at 37°C for 24 h then centrifuged at 16000 g for 30 min. Pellets derived from 1 g of brain tissue were pooled and resuspended in 3 mL of deionized, ultra-pure H_2_O and were dialyzed against three changes of deionized, ultra-pure H_2_O (MWCO 10–12 kDa) over 24 h to remove small molecular weight contaminants.

### Lichen extract reactions

Degradation of PrP by lichen extracts was assessed either using preparations enriched for PrP^TSE^ (PTA-enriched PrP^TSE^) or PrP in a complex matrix of brain materials. BH (10 µL; 1 mg brain equivalent) or PTA-enriched PrP^TSE^ preparation (10 µL; 30 µg total protein) was incubated with 10 µL of lichen extract in DMSO (10 mg of lichen equivalent) and 10 µL of deionized H_2_O at 37°C for 1 h. Control reactions contained 10 µL of DMSO vehicle rather than lichen extracts or contained lichen extract, but were not allowed to incubate (“spiked”). For dose-response experiments employing smaller amounts of lichen extracts, the remaining volume was compensated with DMSO. For experiments investigating the effect of pH on PrP^TSE^ degradation by lichen extracts, 100 mM sodium acetate (pH 5), 100 mM sodium citrate (pH 6) or 100 mM Tris (pH 7 and 8) replaced water in reactions. In enzyme inhibitor experiments, final inhibitor concentrations were: 5 mM ascorbate, 80 mM Cd^2+^, and 1 or 10 mM EDTA. Protease inhibitor cocktail (Sigma-Aldrich, St. Louis, MO) was used at 10 µL per reaction, PefaBloc SC was at 10–14 mM and all other protease inhibitors were at the concentrations listed in Table 1.

For experiments with *P. sulcata* water-extract, 10 µL of PTA-enriched PrP^TSE^ was added to 990 µL of water, lichen water-extract (∼100 mg lichen equivalent) or lichen water-extract in the presence of PefaBloc SC (10 mM) and was shaken for 1 hour at 20°C. Following incubation, protein was precipitated with trichloroacetic acid (TCA) and pellets were resuspended in 100 mM Tris, pH 7.0.

Methods to clean-up samples in an effort to recover PrP immunoreactivity included precipitating 30 µL reactions containing equal parts of PrP^TSE^, water and lichen extract or DMSO with 4 volumes of ice-cold methanol or TCA. Other samples were diluted with 470 µL of PBS and placed in microcentrifuge concentrating/desalting columns (Centricon, 10 kDa MWCO; Millipore, Billerica, MA) and subjected to centrifugation. When sample volumes were reduced, the material on the filter was washed 3 times with 500 µL of PBS prior to recovery of the remaining PrP into a clean microfuge tube. Reactions to be analyzed by immunoblotting were halted by the addition of 4× lithium dodecyl sulfate (LDS) sample buffer and 10× NuPAGE reducing agent (Invitrogen, Carlsbad, CA), each to final concentrations of 1×, and were then heated at 95°C for 5 minutes.

### PK digestion

Samples of PrP^TSE^ were incubated with 20 to 10,000 µg·mL^−1^ PK in 33 mM Tris (pH 7.0) for 1 h at 37°C. Following incubation, digestion reactions were halted by additions of 4× LDS sample buffer and 10× NuPAGE reducing agent, each to final concentrations of 1×, and were then heated at 95°C for 5 minutes.

### Lichen chemical-PrP^TSE^ reactions

The lichen secondary metabolites usnic acid, atranorin (both from Sigma-Aldrich, St. Louis, MO) and lecanoric acid (the generous gift of D. Fahselt) were dissolved in DMSO to final concentrations of 3, 10 or 100 mg·mL^−1^. Compounds were added to PTA-enriched PrP^TSE^ preparation (10 µL) in the presence of 10 µL of deionized, ultra-pure H_2_O and incubated at 37°C for 1 h. Samples were then treated with LDS sample buffer and reducing agent and heated 95°C for 5 minutes.

### Live lichen-BH incubation

Freshly collected lichen tissue was air dried for ≥48 h prior to placing an intact 4 mg portion into 100 µL of dialyzed, HY BH. Lichen-BH samples were incubated at 20°C for 24 h with agitation. Samples lacking lichen tissue served as controls. Following incubation, the BH was removed from the lichen tissue by micropipette and 30-µL aliquots of lichen-treated and control BH were analyzed by immunoblotting. Lichen tissue, after exposure to BH, was ground in 1× NuPAGE sample buffer with reducing agent using a rotary pestle and then heated at 95°C for 5 min. Lichen particles were precipitated by a touch spin in a microfuge while the samples were still hot, and the sample buffer was collected and promptly subjected to NuPAGE and immunoblotting.

### Extraction of PrP from chitin

To assess extractability of PrP^TSE^ from lichens, 10 µL of PrP^TSE^ was added to 100 µL of chitin-coated magnetic beads (New England BioLabs, Ipswich, MA) in a final volume of 1 mL in distilled H_2_O. Control samples lacking beads and chitin bead samples were incubated for 24 h at 20°C with shaking. Beads were removed from solution by magnet and protein in the remaining solutions was precipitated with 4 volumes of ice-cold methanol. Extraction of beads and resuspension of the pellets was accomplished with 1× NuPAGE sample buffer.

### Protein Misfolding Cyclic Amplification (PMCA)

All PMCA procedures were performed according to the method described in Fujihara, *et al.* [68]. Following incubation of RML-infected BH with *L. pulmonaria* extract (for either 1 hr or spiked into the sample for the minimum time possible) or vehicle, each sample was divided into two aliquots; one aliquot was prepared for immunoblotting as described above and one aliquot was serially-diluted in PMCA buffer (150 mM NaCl, 1 mM EDTA, 50 mM HEPES pH 7.0, 1% Triton X-100, 0.05% digitonin and EDTA-free protease inhibitor cocktail; Roche Applied Science, Germany). Substrate for PMCA was the supernatant from previously-frozen, healthy CD-1 mouse brain tissue homogenized to 10% (w/v) in PMCA buffer and centrifuged at 2000 *g* for 2 min. Reactions were held in snap-cap 200 µL PCR tubes and the reaction mixture was composed of 79 µL substrate and 1 µL of seed (e.g., dilutions of *L. pulmonaria* extract-treated, *L. pulmonaria* extract-spiked or vehicle-treated infected BH). Amplification was performed using a deep-well cup-horn Misonix sonicator (Model 3000; Farmingdale, NY) attached to a circulating water supply in an incubator set to 37°C. Sonication for 20 s every 30 min at 60% power was performed for 48 h. As controls, reactions containing substrate without infected BH seed were also subjected to cycling and samples containing substrate and infected BH were held at 37°C without cycling, but neither showed amplification. Following PMCA, samples were treated with 20 µg·mL^−1^ PK, and an aliquot was analyzed by NuPAGE and immunoblotting.

### NuPAGE and immunoblotting

Samples were subjected to 12% NuPAGE gel electrophoresis using MOPS running buffer and electroblotted to polyvinyl difluoride membranes. Membranes were then immunoblotted using monoclonal antibodies (mAb) 3F4 (1∶20000), SAF83 (1∶5000) and SAF84 (1∶200) (Chemicon, Billerica, MA), 6H4 (Prionics AG, Switzerland, 1∶10000) or polyclonal antibody (pAb) rabbit anti-20S proteasome subunit C2 (1 µg·mL^−1^; A.G. Scientific, San Diego, California). Anti-mouse and anti-rabbit secondary antibodies conjugated with horseradish peroxidase were used to detect mAbs and the pAb, respectively (Santa Cruz Biotech, Santa Cruz, CA). Visualization was performed using Pierce (Rockford, IL) SuperSignal West Pico Chemiluminescent Substrate System and an EC3 imaging system (UVP, Upland, CA). For presentation purposes, some irrelevant lanes were excised from images of membranes and no further changes to brightness or contrast were made following excision. Data from separate gels presented in the same figure are separated by a black line or presented in a separate box. All immunoblot results were confirmed by at least three independent experimental replicates done in separate reaction tubes.
